# An Additive Manufacturing Test Artifact

**DOI:** 10.6028/jres.119.017

**Published:** 2014-10-23

**Authors:** Shawn Moylan, John Slotwinski, April Cooke, Kevin Jurrens, M Alkan Donmez

**Affiliations:** 1 National Institute of Standards and Technology, Gaithersburg, MD 20899; 2 Johns Hopkins University Applied Physics Laboratory, Laurel, MD 20723; 3 Commonwealth Center for Advanced Manufacturing (CCAM), Disputanta, VA 23842

**Keywords:** 3D printing, additive manufacturing, benchmarking, performance evaluation, test artifact

## Abstract

A test artifact, intended for standardization, is proposed for the purpose of evaluating the performance of additive manufacturing (AM) systems. A thorough analysis of previously proposed AM test artifacts as well as experience with machining test artifacts have inspired the design of the proposed test artifact. This new artifact is designed to provide a characterization of the capabilities and limitations of an AM system, as well as to allow system improvement by linking specific errors measured in the test artifact to specific sources in the AM system. The proposed test artifact has been built in multiple materials using multiple AM technologies. The results of several of the builds are discussed, demonstrating how the measurement results can be used to characterize and improve a specific AM system.

## 1. Introduction

Additive manufacturing (AM)—also known as additive fabrication, 3D printing, direct part manufacturing, layered manufacturing, and freeform fabrication—is defined as the process of joining materials to make objects from three-dimensional (3D) model data, usually layer upon layer, as opposed to subtractive manufacturing methodologies such as machining. There are many different AM technologies—for example, stereolithography, laser sintering, multi-jet printing, etc.—the ASTM International standard terminology document [1] for AM groups these technologies into seven different process categories: binder jetting, directed energy deposition, material extrusion, material jetting, powder bed fusion (PBF), sheet lamination, and vat photopolymerization. For the purposes of this paper, a machine is defined as the physical structure comprising its components and their motions along with any computer software controlling those motions. The process is the physics involved in producing the part. For example, the process in laser sintering involves the laser beam melting a specific section of a powder bed, fusing particles together to form a part. The processing parameters are the inputs to the machine that govern the process (e.g., laser power in laser sintering). A system encompasses both the machine and the process.

Additive manufacturing research at the National Institute of Standards and Technology^1^ (NIST) focuses on metal-based AM (primarily laser-based PBF) because parts produced by these technologies are likely to be used as functional components, have a higher inherent value than parts made from other materials, and require further improvements before widespread acceptance of metal-based AM can be achieved. However, the need for better understanding of the technologies through system performance characterization is universal.

In manufacturing metrology, two primary approaches exist to evaluate the performance of a system: (1) through a series of direct measurements of system components or characteristics, and (2) through measurements of manufactured test artifact. The former requires positioning and/or control of individual machine components (e.g., an x-axis slide) and measuring instruments mounted in and around the machine’s work volume to measure relative positions, orientations, velocities, etc. of these machine components. This is often difficult or impossible with AM systems, either because the moving components are not accessible to the end user or safety controls for potential hazards (e.g., high-power lasers) prevent the user from operating the system with the measuring instruments in the way. While research and development of measurement methods for direct characterization of AM machines is ongoing, the better approach for performance characterization, at least in the short term, is through test artifacts.

Manufacturing a test artifact enables a composite test since most errors in the system combine to contribute to errors in the part. The disadvantage of composite tests is that linking specific part errors to specific system error sources is often difficult. However, the advantages of test artifacts are that producing parts is directly aligned with the actual purpose of the system and specialized measuring equipment is typically not necessary since the required equipment is common for discrete part manufacturing.

The primary purpose of a test artifact is to quantitatively evaluate the performance of a system. The clear benefit of a standard artifact is that different systems that produce the same standard artifact can be easily compared. Additionally, if designed properly, the standard test artifact can test the limitations of the system. The standard test artifact can serve as a method for performance verification between system users and vendors, as well as provide a platform for vendors to demonstrate improvements in their AM systems.

This document details NIST efforts in developing a test artifact for AM. First, “rules” for test artifacts are discussed. These rules are drawn from existing literature as well as experience in working with test artifacts for different technologies. A review of existing test artifacts used to characterize AM systems and technologies that are reported in the literature follows. Details of the design of a test artifact for AM intended for standardization are presented before discussing the measurements taken on the test artifact. Results obtained from building the proposed artifact on several AM systems are discussed. Finally, a strategy for standardizing a test artifact for performance characterization of AM systems is discussed.

## 2. “Rules” for Test Artifacts

Additive manufacturing gained prominence in the late 1980s and early 1990s and was commonly referred to as layered manufacturing, three-dimensional printing (3DP), or rapid prototyping (RP). Stereolithography (SLA™) was the first commercial additive RP technology, followed closely by fused deposition modeling (FDM™), laminated object manufacturing (LOM™), and selective laser sintering (SLS™). As AM matured, many more technologies, such as material jetting, entered the market. Early in the development of stereolithography, Richter and Jacobs [2] saw the need for a standard accuracy test to help provide quantitative results and noted the qualities of an “ideal accuracy test part.” Paraphrasing, the standard test artifact would:

- be large enough to test the performance of the system near the edges of the platform as well as near the center,
- have a substantial number of small, medium, and large features,
- have both holes and bosses (protruding features) to aid in verifying beam width compensation,
- not take too long to build,
- not consume a large quantity of material,
- be easy to measure, and
- have many features of a “real” part (e.g., thin walls, flat surfaces, holes, etc.).

Other researchers [3] have followed these criteria/rules closely. Byun [4] referenced these rules, but added that the test part should include features along all axes and should include features used to determine the minimum feature size attainable.

While many of these qualities are indeed important considerations in designing a test artifact, an ideal artifact would not only reveal most errors and limitations of a system, but it would also correlate those errors and limitations with specific aspects of the sytem. Kruth [5] moved in this direction, noting that a test artifact should not only evaluate system limitations, but should also include features to allow iterative processing parameter optimization. Scaravetti [6] took this idea a step further, stating that the qualification procedure must make it possible to identify and quantify defects, but also determine the sources of the defects. In order to do this, the test artifact should:

- have simple geometrical shapes, allowing perfect definition and easy control of the geometry,
- require no post-treatment or manual intervention (e.g., there should be no support structures), and
- allow repeatability measurements.

In addition, several researchers state or imply the need for a test artifact to include multiples of the same feature to allow repeatability measurements. However, including multiples of the same feature merely tests the system’s capability to produce that same feature at different places within the build volume; it does not test the repeatability of the system [7]. Since various conditions may result in different systematic errors at different locations in the build volume, this leads to differences in the shapes of features produced in these positions. Therefore, if multiple artifacts were produced by a system with perfect repeatability, features produced in the same position in the build volume would be exactly the same, but they still may be misshapen.

While there are a multitude of test artifacts for AM proposed in literature (further discussed later), not all of them abide by the guidelines discussed above, and none of them have gone through a formal standardization procedure. However, one example of successful implementation of a standardized test artifact can be found in the area of machine tool metrology.

## 3. Circle-Diamond-Square

**Fig. 1:**
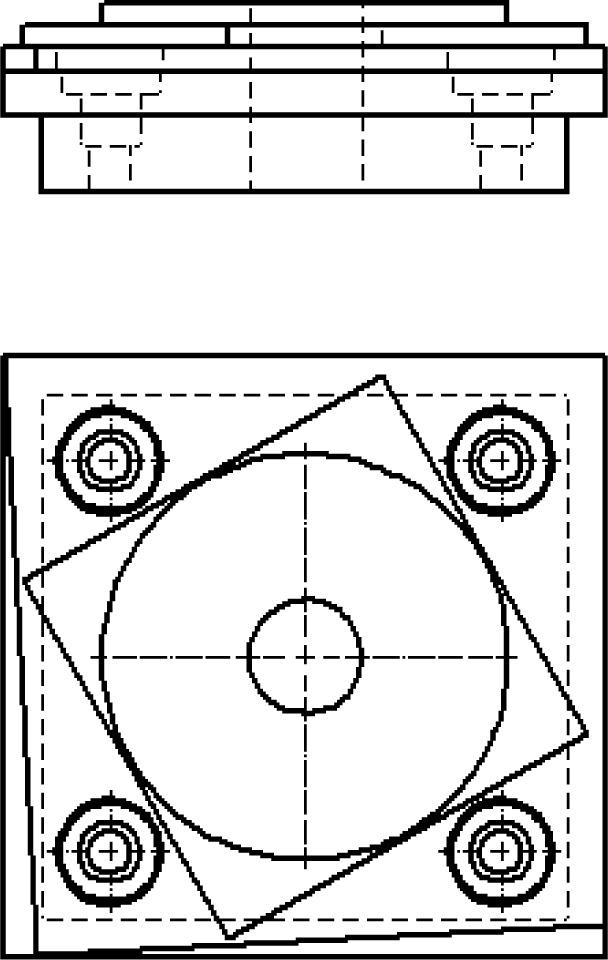
Test artifact, commonly known as the “circle-diamond-square,” for machining centers described in Refs. [8–10].

The use of test artifacts for performance characterization is not unique to AM, and in fact, several standard artifacts are used to characterize metal cutting machine tools (see, for example, Ref. [8] and Ref. [9]). One example of a test artifact that benefits U.S. manufacturers is the standard machining test artifact known colloquially as the circle-diamond-square test piece. This test piece is described in Ref. [8] and Ref. [9] (and is shown in Fig. 1), but is well established in the machining community, having been originally defined in 1969 in Ref. [10].

The strength of the circle-diamond-square artifact lies in its simplicity. Each feature of this artifact tests a specific aspect of the machining center used to produce it. The outside square tests the straightness of the individual axis used to cut the respective side of the square and the squareness between the two axes. The diamond feature tests the ability of the controller to execute linear interpolation of two axes. The large circle feature tests the ability of the controller to execute circular interpolation of two axes. The four pairs of concentric circles are measured for true position and characterize the machine’s linear positioning accuracy. The small angle cuts test the machine’s smallest actionable step. The drilled hole in the center tests the performance of the machining center’s main spindle. As such, errors observed in a specific feature can often be attributed to a specific aspect of the machining center’s performance. Additionally, the test part is easy to design in computer aided design (CAD) or computer aided manufacturing (CAM) software, and machining processing parameters are suggested within the standard that defines the part. The test part is easy to measure, and several measurement options exist in addition to measurement with a coordinate measuring machine (CMM).

An interesting aspect of this test part, as defined in Ref. [9], is that tolerances are associated with the part. These tolerances were established by the International Organization for Standardization (ISO) standards committee through the consensus of representatives from machine tool builders, machine tool users, government institutes, and academia. The tolerance values are intended to be met or exceeded by a relatively modest (not high-end) machine tool. It is noteworthy that the current standard tolerance levels for machining of this test part are on the order of 0.020 mm, likely tighter than most metal-based AM system capabilities [11]. By setting a baseline for system performance, system builders can often demonstrate quantitatively the extent to which their systems exceed standard specifications.

One consideration in the design of the machining test artifact is that metal cutting machine tools were well established when the standard test artifact was developed in 1969. Most metal cutting machine tools (especially three-axis machine tools) have a similar design of stacked linear stages. Since most machine configurations are similar, the observed errors and limitations are similar. As such, it is easier to design a standardized test part with features that reveal these common errors and limitations and to make it possible to compare the performance characteristics of various systems.

Additive manufacturing technologies and systems are less mature and several different machine configurations exist to correspond with different types of processes. These differences have led to a number of proposed AM test artifacts to-date, many of which have very different features and designs.

## 4. Additive Manufacturing Test Artifacts

Based on a substantial review of the available literature, the following sub-sections briefly discuss many of the different test artifacts used to characterize different aspects of the performance of additive systems. The resulting test artifacts are described in four categories: test artifacts for comparing systems or technologies for decision making, test artifacts for evaluating individual systems for optimization, test artifacts for evaluating metal-based processes, and test artifacts for other uses. This discussion is by no means exhaustive, nor is it unique.

### 4.1 Test Artifacts for Comparing Systems and Technologies for Decision Making

**Fig. 2:**
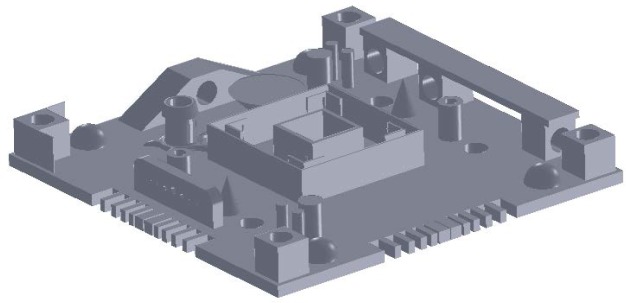
Solid model of the test artifact used by Mahesh [21]. Ramps, cones, hemi-spheres, and overhanging features are all present.

Many researchers proposed the use of test artifacts, often called benchmarking parts, to quantitatively compare the capabilities of the various systems or technologies [4, 12–24]. This was especially the case early in the development of RP technologies; users wanting to benefit from the advantages of RP had to choose which of the technologies best fit their application, and comparative studies with benchmarking parts aided in that decision making. Kruth [12] was the first to mention a test artifact for comparing AM systems, citing a study done by two Dutch companies using a U-shaped artifact with various geometric features such as circular holes (in various orientations), circular bosses, square holes, and angled surfaces. Other researchers built upon these results, adding more and/or different features, including overhangs and freeform objects, to demonstrate some of the advantages of additive technologies (see Fig. 2) [14, 21]. Still more researchers investigated the surface roughness of test parts in addition to geometric accuracy as a means to compare AM systems [16, 17]. As additional AM technologies gained prominence, researchers added these to the comparative studies. For example, Byun added 3DP [4] and Kim added material jetting [23]. Ultimately, specific AM technologies gained sufficient capability and acceptance to support multiple machine platforms and manufacturers, leading to comparative studies of systems within a process category, e.g., seven types of 3DP systems produced by six different manufacturers [19, 20].

### 4.2 Test Artifacts for Evaluating Individual Systems

**Fig. 3:**
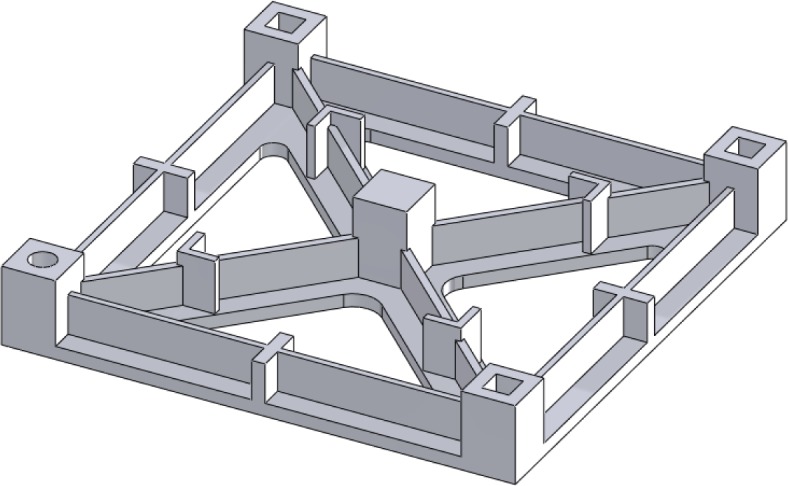
Solid model of the “user part” [25] that focuses on assessing the machine accuracy in the x-y plane.

Artifacts are also used to evaluate individual systems, either when a new process or material emerges in the market [25–32] or when system improvement/optimization is the goal of the study [3, 33]. The so-called “user part” is one of the first test pieces designed to quantitatively assess the accuracy of stereolithography systems (see Fig. 3) [25]. This part was designed in 1990 by an SLA user group and focuses on assessing the machine accuracy in the x-y plane. This same part, or slight variations of it, have been used by many other studies to characterize other additive technologies, including evaluation of laser sintering for indirect manufacturing of metallic components (i.e., using laser sintering to create an intermediate, “green” part that must undergo subsequent post processing to become fully dense) [27]. Additionally, when new material options are introduced into an established technology, it is important to quantify the accuracy of parts made with this new material [30, 32].

Development of new materials led to the first test artifacts dedicated to process improvement and optimization. During stereolithography’s first years, newer, stronger materials were being quickly developed and introduced into user systems. The “windowpane” and “Christmas tree” test artifacts were used at this time to quantify the effects of changing various processing parameters, leading to iterative optimization [33].

Examining the literature for use of test artifacts to evaluate individual systems may lead to the incorrect conclusion that using a test artifact to optimize processing parameters is uncommon. In reality, most AM system manufacturers and users working on system development typically have their own internal test pieces for this purpose, though their designs may not appear in publically available literature.

### 4.3 Test Artifacts for Evaluating Metal-Based Technologies

**Fig. 4:**
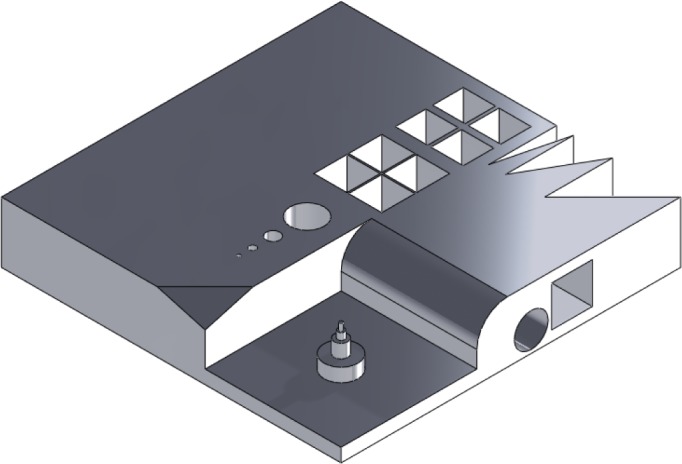
Approximate re-creation of the test artifact described by Kruth [5] to determine geometric errors and surface roughness, and to allow for the extraction of mechanical testing specimens.

**Fig. 5:**
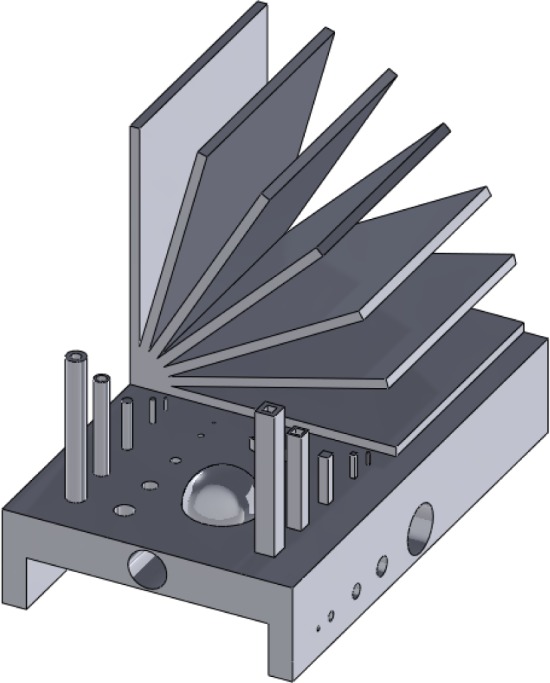
Approximate re-creation of the test artifact used by Castillo [34].

**Fig. 6:**
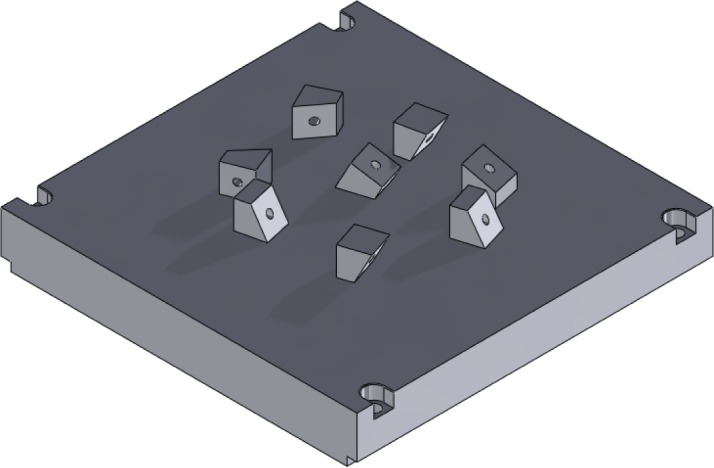
Approximate re-creation of Delgado’s simple artifact in various orientations on an AM build platform [39].

In recent years, the capabilities of metal-based AM systems have grown tremendously and these systems have emerged as viable methods for the direct manufacturing of metallic parts (i.e., using an energy source to melt and bond metal raw materials, creating a fully-dense part without the need for substantial post-processing). Numerous studies have concentrated on test artifacts to evaluate metal-based AM technoloogies [5, 11, 34–39]. The various technologies that have been studied include selective laser sintering (SLS™), selective laser melting (SLM™), direct metal laser sintering (DMLS™), electron beam melting (EBM™), and micro-welding technologies. Kruth [5] took a traditional approach by creating a test artifact with characteristic features to determine and analyze geometric errors and surface roughness (see Fig. 4). Additionally, this artifact included features to be extracted for mechanical testing to provide information about mechanical properties. The study used other features of the artifact to determine the capabilities and limitations of the different AM systems used for fabrication. Castillo [34] provided a similar comparison, though with an innovative test artifact that characterized the system’s accuracy and capability to build at various angles using an “open book” feature (see Fig. 5). Ning [35] investigated the process characteristics of DMLS™ using test artifacts that examine the effects of part shrinkage during the building, which is minimized through a shrinkage compensation function. Ghany [36] used a “real-world” test part as the reference (instead of fabricating an arbitrary design) and compared the visual appearance, mechanical properties, chemical composition, microstructure, and production costs of the components made by different metal-based AM technologies. Hanumaiah [37] manufactured numerous benchmark parts to assess geometric errors of the various features incorporated in the designs. Similarly, Pessard [38] created a test part used to evaluate the dimensional accuracy of embedded features. Delgado [39] followed with another test part used to assess geometric accuracy, but this structure was fabricated in many locations and orientations throughout the build platform in an attempt to evaluate the accuracy and repeatability of position (see Fig. 6). Finally, Cooke used a common machining test artifact to assess the geometric accuracy of its features when built using two different metal-based additive technologies [11]. Differences in the processing parameters, however, prevented comparative conclusions from being drawn.

### 4.4 Test Artifacts for Other Uses

The primary purpose of most test pieces previously mentioned was to characterize the accuracy of systems. Other researchers have used test artifacts to examine different aspects of AM. The layer-upon-layer nature of AM typically leads to stair-stepping on sloped and freeform structures based on the layer thickness. Accordingly, several research efforts have focused on the surface roughness of various additive technologies [18, 40, 41]. Similarly, the layer-upon-layer nature of AM can lead to unique and often anisotropic mechanical properties of materials, triggering further investigations of these properties through producing test artifacts [42, 43].

## 5. Summary of Part Designs

While all of the AM test artifacts mentioned in this report are different, many commonalities exist. Common aspects are to be expected because much of the research builds upon the findings of previous work, and many researchers were influenced by the “rules” put forth by Richter and Jacobs [2]. Most of the test artifact designs have various “real” features atop a square or rectangular base (see Figs. 2–5). The various features observed are:

- rectangular holes, bosses, and tubes (in multiple directions),
- round holes, bosses, and tubes (in multiple directions),
- spherical holes and bosses,
- conical bosses,
- L-shaped bosses,
- ramps,
- overhangs,
- angles,
- side notches,
- thin walls and fine features,
- freeform structures, and
- towers.

The sizes of the test artifacts varied, but the largest observed dimensions of the square base were 240 mm by 240 mm. The smallest features observed were 0.25 mm thin walls (for both polymer-based and metal-based AM technologies), 0.2 mm holes and bosses in polymer-based AM technologies, and 0.5 mm holes and bosses in metal-based AM technologies.

One alternative to the square-base, multiple-feature test artifact is the use of a smaller, simpler test artifact that is built at multiple positions and/or multiple orientations in the build volume (see Fig. 6). A second alternative approach is the use of a standard library of 3D objects or part features (e.g., spheres, cylinders, prisms, cones, etc.), rather than a single test artifact, to evaluate AM system performance. Smith proposes a library of twelve objects to benchmark AM systems, with each object designed to demonstrate and evaluate at least one important feature of the resulting parts [44]. Jurrens suggests use of a standard library of 3D features that would be built and measured in a standard way [45]. The standardized features would be built in a variety of sizes, locations, and orientations, and potentially would be supplemented with selected “real-world” parts.

## 6. Design Criteria

Previously, the concepts of using a test artifact to highlight the capabilities and limitations of a system or to identify and quantify specific system errors have remained mostly distinct. The current study seeks to combine the concepts into a singular artifact and draw from the strengths of previously designed test artifacts both in the field of AM and elsewhere.

The selection and location of features in a test artifact are chosen based on the intent of the design. The intent of most designs is to characterize the accuracy of the system or to demonstrate the capabilities of the system. We seek to design a test artifact that is capable of highlighting the capabilities and limitations of a system, quantifying the accuracy of a machine, and diagnosing specific errors in the system. Further, we seek a design, like the circle-diamond-square artifact, whose features all serve a specific purpose, are simple in design, and are easy to measure with low measurement uncertainty. These simple features reduce the likelihood of errors in the design files and increase the likelihood that the proposed artifact can be built using as many current and future AM technologies as possible. The following subsections explain criteria for demonstrating capabilities and limitations of a system, criteria for identifying and quantifying system errors, and some general considerations. A summary of the part and system capabilities of interest is given at the end of the section.

### 6.1 Criteria for Demonstrating Capabilities and Limitations

To design an artifact that demonstrates capabilities and limitations of a system, certain “required” AM system capabilities must first be established. These capabilities should indicate that the system is (or is not) capable of producing a desired “real world” part, and should also show unique aspects of the system. An AM system should be able to create features with proper form (straight features, circular and arced features, etc.) and orientation (parallel and perpendicular features). The system should build features that have correct size, and it is desirable to know the smallest-sized feature the system can create. The system should be able to produce these features as both holes (cavities) and bosses (free standing structures). It should be able to create these features not just in the horizontal plane (parallel to the build platform, the x-y plane), but also in the vertical planes as well (i.e., overhangs). Finally, the system should be able to produce these features in the correct locations.

### 6.2 Criteria for Identifying and Quantifying System Errors

Establishing criteria for highlighting capabilities and limitations of AM systems that can be generalized for all systems is simplified because the focus is on the final part. Conversely, establishing criteria for identifying and quantifying system errors is complicated not only because of the difficulty in separating machine characteristics from process characteristics, but also because the focus is on the machine itself and different AM technologies often use dissimilar machine setups. For example, processes that rely on laser beams often position the beam spot by deflecting the beam using two mirrors that may be independently rotated, while processes that rely on binder jetting often position the binder using a slow carriage, an orthogonal fast carriage, and an array of binder nozzles. The structures and movements of these different systems do not bear much resemblance to each other. However, a couple of general concepts exist that allow for the use of a common artifact to test the different systems. First, all systems generally comply to the use of x,y,z- coordinate axes as established by international standards [46, 47]. Second, a feature is produced by positioning a certain machine component (whether it be an energy source, nozzle, or something else) relative to the position of the build platform.

With these concepts in mind, the primary focus of NIST research is on metal-based additive technologies, and the current primary in-house technology is a PBF-laser system. Several characteristics in a PBF system are critical for accurate part production, including:

- errors in positioning of the laser beam (resulting from geometric errors of the two axes of rotation holding the laser beam positioning mirrors or form errors in the f-θ lens that focuses and shapes the laser beam spot),
- geometric errors of the axis positioning the build platform,
- alignment errors between the axes,
- errors in the laser beam size and shape,
- variation in the beam power,
- irregular powder size distribution,
- error in powder flatness.

Of these, powder distribution and flatness, along with beam power and shape, cannot be tested through post-process measurement of a test artifact. The other machine characteristics can be tested this way, and therefore the test artifact should be designed in a way to identify and quantify these characteristics.

### 6.3 General Considerations

Of course, a test artifact that satisfies the above criteria but is ultimately impractical to build and measure will be of no real benefit. As such, several additional general characteristics are adopted. First, the test part must be easy to measure with low measurement uncertainty using commonly available measuring equipment (e.g., CMMs, 3D scanners, surface profilers, optical microscopes, digital cameras, etc.). Also, there is a trade-off between testing the entire build volume and not consuming too much time and material in the build. While a balance of the two is preferred, faster, smaller builds tend to win out because of the relatively high cost in both time and material of PBF-laser builds. A design that minimizes other variables such as support structures, post processing, and errors due to file format and file conversions is also desired. The test artifact should allow for various tests of mechanical and physical properties in addition to geometrical accuracy. Finally, the part design should minimize mechanical impact between the powder spreading mechanism (e.g., a recoating blade) and the top surface of each layer.

## 7. The Part

**Fig. 7:**
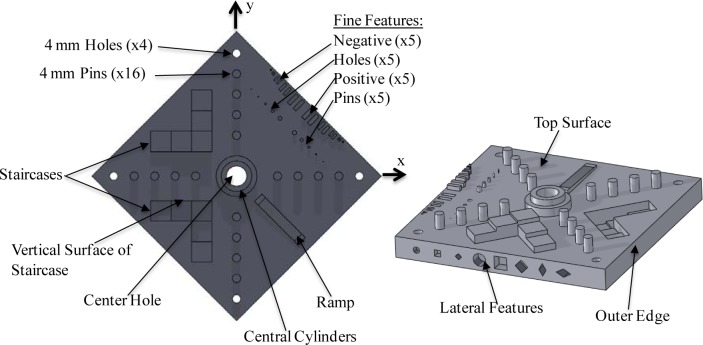
Solid model of the proposed test artifact showing a top view (left) and an oblique view (right) with arrows pointing to important features.

**Fig. 8:**
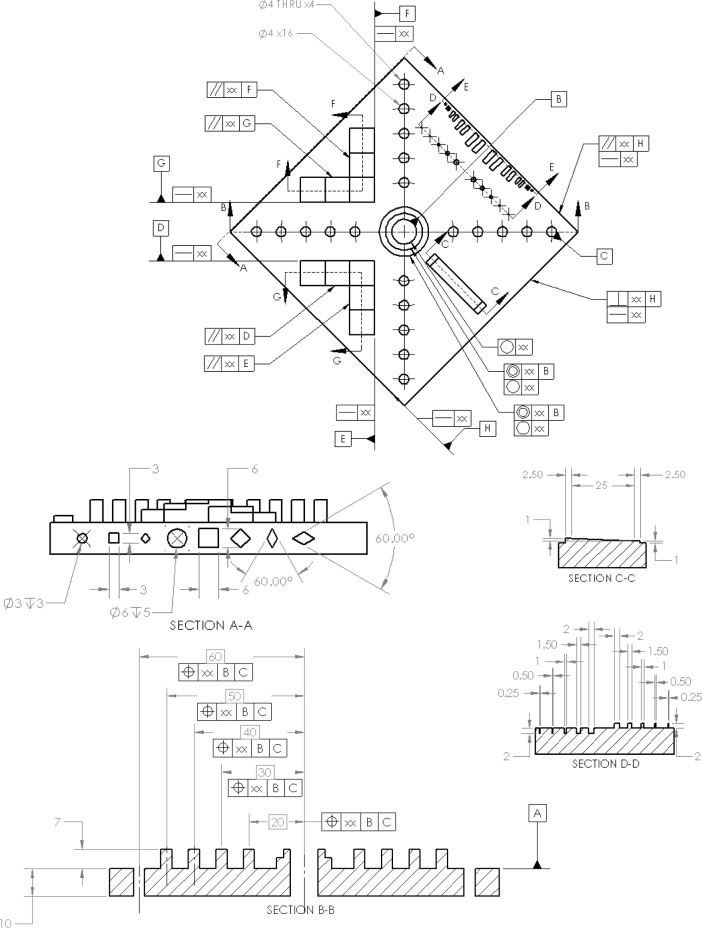

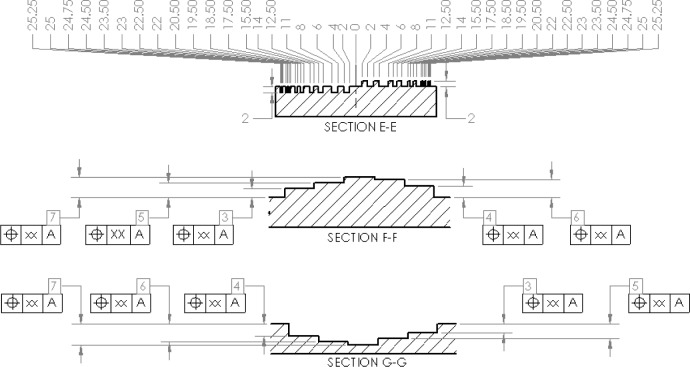
**(part 1).** Engineering drawing of proposed test artifact; all dimensions are in mm. **(part 2).** Engineering drawing of proposed test artifact; all dimensions are in mm.

Based on the design criteria presented in the previous section, NIST has developed a test artifact to be proposed for standardization. A schematic of the current version of this artifact is shown in Fig. 7 and an engineering diagram is shown in Fig. 8. This design is the result of several iterations of building, measuring, and modifying artifact designs to alleviate various problems. The labels in Fig. 7 and the geometric dimensioning and tolerancing (GD&T) symbols in Fig. 8 indicate the important features to be evaluated and the post-process measurements to be taken. Note that tolerance values in the GD&T symbols are marked as “xx” because the test artifact itself does not have a tolerance, but the results of measurements on these features will help establish the ability of the system to meet a specified tolerance. The artifact is comprised of several features with simple geometry, atop or within a diamond-shaped base. Simple geometries were chosen to simplify measurement and minimize the likelihood for errors in the design file (note that the geometric fidelity of the design file has not been examined). Each of these features is discussed in detail in the following subsections and the measurements to be taken on the features are discussed in the following section. Table 1 summarizes the features and characteristics investigated by the various features of the test artifact.

The part should be built in the center of the building platform with the 4 mm pins and holes aligned along the machine x- and y-axes. It is 17 mm tall and has a volume of approximately 101 000 mm^3^. The diamond shape was chosen because it will minimize the impact between the recoating blade and each layer of the part, and it allows simple vertical mounting for various measurements.

### 7.1 Top Surface

The top surface of the part is the primary datum feature defining the z-direction and the z-origin of the measurement coordinate system. This feature characterizes the ability of the AM system to make flat, smooth upwardly-facing surfaces.

### 7.2 Center Hole

The center hole in the part is the secondary datum feature defining the x- and y-origin of the measurement coordinate system. This feature characterizes the system’s ability to produce round holes. Additionally, the perpendicularity of this hole relative to the top surface indicates the alignment of the z-axis positioning system (i.e., the movement of the build platform) to the x-y plane. The cylindricity of this hole (or straightness) characterizes the straightness error of the z-axis motion.

### 7.3 Pins and Holes

The right-most hole in the top view of the part is the tertiary datum feature defining the x-direction of the measurement coordinate system.

Ideally, the motion of only one positioning axis would control the location of the pins and holes aligned with the respective machine axis direction. This would be the case for machines comprised of orthogonal linear axes (e.g., material extrusion machines). This might also be the case for many energy beam systems utilizing mirrors to position the beam if the mirrors are orthogonal to each other and aligned to the machine axes, and the part is directly below the beam (i.e., the beam is perpendicular to the part’s top surface at the center of the part). This is likely not the case for DMLS machines because they often utilize a non-orthogonal mirror orientation. However, even if multiple machine axes need to move to locate the positions of the pins and holes, the systems still utilize the concepts of x- and y-axes, and the machine is likely compensated using these directions.

With the pins and holes aligned to machine axes, the deviations in the positions of the pins and holes correspond to errors in positioning (or geometric errors) of the respective linear axis. Deviations in the x-direction of the pins aligned with the x-axis are a result of linear displacement error of the x-axis. Deviations in the y-direction of the pins aligned with the x-axis are a result of straightness error of the x-axis. Deviations in the y-direction pins lead to similar conclusions. The linear displacement errors can be compensated in many systems.

**Fig. 9:**
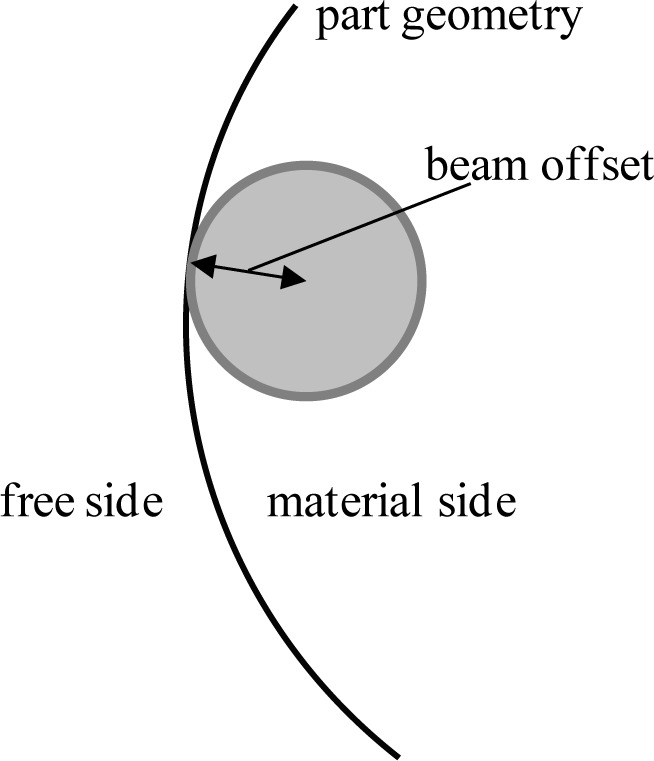
Beam offset can give a good approximation of beam spot size.

These pins and holes can also be used to determine the beam width in energy beam technologies. The machine controller positions the beam center. If the controller commanded the beam center to the edges of the feature’s geometry, the parts would be too large because the beam would fuse a small amount of powder on the free side of the geometry equal to the radius of the beam spot. To compensate for this, the controller uses a beam offset that positions the beam center toward the material side of the feature by an amount equal to the beam radius (see Fig. 9). Therefore, the value used for the beam offset provides a good approximation of the beam size, and if this value in the controller is incorrect, feature sizes will likely be incorrect. To examine the beam offset, the average diameter of the pins can be compared to the average diameter of the holes and to the nominal diameter. If the pins are smaller than nominal and the holes are larger than nominal, the beam offset is too large. If the pins are larger than nominal and the holes are smaller than nominal, the beam offset is too small. Users usually have the ability to tune values for beam offset.

### 7.4 Staircases

The staircase features can be used to highlight several characteristics of the system. The z- positions of the upward facing surfaces of the steps demonstrate the linear displacement errors of the machine’s z-axis (note that shrinkage may also play a role in the features’ deviations in position, but its effect on any compensation would be the same as a deviation purely resulting from linear displacement error). The selection of layer thickness may influence the z-positions of the tops of the steps, especially in cases where the distance between steps (1 mm) is a non-integer multiple of the layer thickness. The vertical surfaces of the staircase features are parallel to the machine’s x- and y-axis directions. Therefore, the long surfaces help to characterize the capability of the system to produce straight features parallel to the machine axes. The shorter surfaces help to check the ability of the system to make parallel features paraxial with the machine axes.

### 7.5 Outer Edges

The three outer edge features that do not contain lateral features characterize the system’s capability to produce straight features and parallel and perpendicular features askew from the machine axes. Also, these features assess the ability of the system to make smooth vertical surfaces.

### 7.6 Central Cylinders

The central cylinders characterize the ability of the system to produce round or arced bosses, as well as the system’s ability to produce concentric features.

### 7.7 Ramp

The ramp is designed to slope constantly with a 1 mm rise over a 25 mm run. However, the discrete layer thickness of any AM system will result in a stair-step effect on the manufactured ramp. The value of the designed slope was chosen so that even systems with small layer thicknesses will still produce the ramp feature with visible stair-stepping. For example, a system utilizing 20 μm layers will produce a ramp with 0.5 mm step landings. The stair-steps demonstrate the minimum actionable design feature attainable in the z-direction and will help characterize the system’s ability to produce 3D contours. Additionally, the z-positions of individual stair-steps can be examined to give further characterization of the machine’s z-axis linear displacement error. The ramp feature has 2.5 mm flat landings at its top and bottom to act as datum surfaces during measurement.

### 7.8 Lateral Features

Whenever possible, lateral features in the test artifact should be produced without support structures. These features were designed with small lateral depths so that a failure to properly build the feature would not result in a complete failure of the build. While a lack of support may result in poor geometry of these features, their failure often reveals important information about the performance of the system. Failure to properly build these features in a PBF-laser system is more often due to poor heat transfer rather than poor structural support, evidenced by singeing at the upper surfaces of these features [48]. Further, if some of the lateral features are well-built without support structures, and others are poorly built without support structures, a user can better understand design rules for when to use supports (e.g., the different shaped diamonds can help provide rules for the minimum draft angle requiring support).

The intent of the test artifact makes support structures problematic for several reasons. From a system characterization point of view, the requirement for the post-process removal of support structures adds ambiguity to the source of deviations in feature geometry, because it may be difficult to de-couple errors that result from the AM system and errors that result from the post-process removal technique. Additionally, from a standardization point of view, a strategy for defining support structures would need to be unambiguous and universally available to ensure all users properly employ it. If support structures are absolutely necessary, the user should fully document the strategy for support (e.g., solid supports, hatched supports, drawn supports, soluble material, etc.) and the geometry of the support structures (e.g., size of the support structures, teeth, or fragmentation).

The resulting geometry of the lateral features combine with the results of the ramp feature to better characterize the ability of the system to produce 3D contours. For example, by examining build quality of the diamond shaped lateral features and combining the severity of the stair-stepping of the ramp feature, a user can make a more informed decision on the ability of the system to produce a “real world” feature like a threaded hole.

### 7.9 Fine Features

There are two different sets of fine features. The first set (closer to the outer edge of the part and shown in section EE of Fig. 8 (part 2)) is of neighboring rectangular bosses or holes. These features help to establish the minimum required separation of features as well as the minimum size of rectangular holes and bosses. The second set of fine features is comprised of the pins and holes shown in section DD of Fig. 8 (part 1). These features are intended to establish the minimum feature size achievable by the system. In both sets, the widths of the fine features descend from 2 mm to 0.25 mm from the center of the set outward. These sizes were chosen in an attempt to ensure the AM system would be able to produce a feature of at least one size and not produce a feature of another size. Surveying capabilities of AM systems, most can produce features that are 2 mm in size. However, few systems (especially metal systems) are capable of producing features that are 0.25 mm in size. As such, a system would be capable of producing a feature on one particular size, but not capable of producing the feature that is the next size down. The minimum feature size attainable would lie somewhere between those feature sizes. All features are 2 mm tall or deep.

### 7.10 Summary

Table 1 summarizes the characteristics of the system being investigated by the various features of the test artifact.

## 8. Measurement

The test artifact should be allowed to cool to room temperature and then measured directly after it is removed from the system used to build it, before any post-processing is performed. Any post-processing performed before measurement makes the results ambiguous because it may not be easy to separate any deviations from nominal that result from the AM system from deviations that result from the post-processing. For example, if the test artifact is built fused to a build platform, measurement should be conducted before separating the test artifact from the build platform because the separation process can result in deformation of the test artifact as a result of residual stresses. If post-processing is desired, measurements should be taken before and after each post-processing step, reporting all measurement results and details of each post-processing step.

The time required to complete the build of the test artifact should be noted. Discussion with AM system users and builders alike indicate that build rate is an important metric for a system. However, there is no current standard definition for build rate. Laser beam or axis feed rates, time to build one layer, and manufactured volume per time have all been used to describe the build rate. These make build rate specifications vague or ambiguous. The time required to build a standardized test artifact is a much more well defined (though not perfect) quantification of build rate.

Most of the prescribed measurements focus on the geometry of the as-built test artifact. The surface roughness of upward facing and side facing surfaces are also easily measured. Additionally, the test artifact has been designed to allow non-destructive evaluation (NDE) to check for porosity as well as the extraction of testing specimens for physical and mechanical property testing. Further testing of various aspects of the part is available, though not all are detailed here.

### 8.1 Measurements Taken on Each Feature

The required measurements can be deduced from the GD&T in Fig. 8 and are summarized below. It is important to remember that GD&T is a product specification only; it is not a prescription for measurement. The measurements described below can be accomplished by a variety of techniques and devices (e.g., coordinate measuring machine, optical scanner, dial indicators with calibrated motion devices, surface profilometers, etc.). Some specific examples of measurement strategies are discussed in more detail below.

#### 8.1.1 Top Surface

The flatness as well as the roughness of the surface is measured.

#### 8.1.2 Center Hole

The cylindricity of the center hole should be measured. Alternatively, the roundness of the center hole along with the straightness of the bore in both the x- and y-directions can be measured. The perpendicularity of the cylindrical hole with the top surface should be measured.

#### 8.1.3 Pins and Holes

The x- and y-positions of each pin and hole relative to the center hole should be measured along with the diameter of each of the pins and holes.

#### 8.1.4 Staircases

The z-position of the upward-facing staircase flats should be measured relative to the top surface. Further, the long vertical surfaces of the staircase are measured for straightness and the short vertical surfaces are measured for parallelism with their associated long faces. Additionally, the perpendicularity of the two long faces of each staircase can also be measured. If the top surface is inaccessible for surface roughness, the surface roughness on the upward-facing surface of the top step in the positive staircase can be measured.

#### 8.1.5 Outer Edges

The three outer edges that do not contain the lateral features are all measured. The primary outer edge (datum feature H in Fig. 8) is measured for straightness. The parallelism of the face opposite the primary outer edge and the perpendicularity of the face adjacent to the primary outer edge are both measured. The distance between the primary outer edge and the opposite face can also be measured. Further, the surface roughness of the outer edges can be checked in both across the individual layers (in the z-direction) as well as parallel to the individual layers.

#### 8.1.6 Central Cylinders

The roundness of the outer surfaces of both the inner and outer cylinders is measured. Further, the concentricity of the inner cylinder to the central hole and the concentricity of the outer cylinder to the central hole are also measured.

#### 8.1.7 Ramp

The ramp is measured to inspect the z-distances between the inevitable individual steps. This can be accomplished using any measurement system designed to measure surface profile. A stylus profilometer was preferred because it was capable of measuring the profile over the entire length of the ramp in one measurement. Measurement by optical profilometer would be equally acceptable, but several individual steps should be measured, which may require multiple measurements.

It should be noted that the measurement of the ramp is for surface profile, not for roughness. A surface profile is the raw data that is filtered and converted to provide roughness results. However, in this case, the surface profile should not be filtered and should be used directly to provide a quantitative measurement of the step heights. The output (and therefore the significance of the measurement) for this feature is intended to indicate the minimum actionable design feature for the AM system under investigation.

#### 8.1.8 Lateral Features

Quantitative measurement of the lateral features may be difficult to attain for certain AM systems. The difficulty stems from the fact that with some AM technologies (e.g., DMLS) the as-built part is fused to a build platform. Depending on the size of the build platform as well as the size and configuration of the measurement device, these lateral features may be inaccessible to the measurement device without removing the test artifact from the build platform, and the issues presented by post-processing of the test artifact before measurement have been previously mentioned. In this case, the lateral features may be evaluated qualitatively through simple observation.

If the test artifact is built without being attached to a build platform, certainly a CMM can be used to measure the diameter and roundness of each of the cylindrical holes, as well as the height and the width of the square holes.

#### 8.1.9 Fine Features

The measurement of the fine features is intended to be observed under optical microscope. The small sizes of the features inhibit access by a typical CMM probe or the line of sight of an optical scanner. Visual inspection by microscope does not provide a value for dimensional accuracy (as in measurement by CMM), but the microscope images (usually at no more than 5x magnification) give an adequate indication of whether or not the feature was successfully built. The fine features of the first set are located close to the edge of the part so that the part can be oriented vertically, and these features can be inspected in profile (if the top view of the features is ambiguous). The final result should be a successfully-built feature of one size and an unsuccessfully-built feature at the next smaller size. This provides a range within which resides the actual minimum feature size attainable. Note that an optical CMM or a calibrated optical microscope could provide dimensional accuracy of the features as well.

### 8.2 Measurement Strategy

It is well known that measurement strategy affects the overall measurement uncertainty; this is true for dimensional measurements and surface measurements alike. Measurement strategy, here, involves the device chosen to perform the measurement along with the number of points selected to represent the feature or surface and the distribution of points along the feature or surface. For roughness measurements, the measurement strategy includes the cutoff length. Measurement strategy is a complicated subject and is often very specific to the part or feature being measured. As such, there is no general “best practice” for performing these measurements. However, the measurement uncertainty is ultimately the important concept, and, considering the available measurement devices, using a measurement strategy that minimizes the measurement uncertainty should be the primary focus of the user. Geometry was measured using a CMM, surface roughness using a stylus profilometer, and fine features using an optical microscope.

#### 8.2.1 CMM

In general, a larger number of points distributed over the entire feature being measured provides a lower measurement uncertainty. A number and distribution of points was selected for each measurement that was greater than the minimum number of points required and also allowed measurement to be conducted in a practical length of time. The example discussed here is included for completeness and should not be considered as a best practice. Twelve points were used to measure the flatness of the top surface; eight of the points were near the outer edges of the part, and four of the points were nearer the center of the part. Three levels with eight points each were used to measure the cylindricity of the center hole. The pins and holes were all measured with 6 points, 3 mm from the top of the feature. One point was used to measure the height of each stair step. Straightness measurements were conducted using one line segment with at least 15 points distributed over at least 80 % of the feature’s length. At least 10 points, also distributed over a line at least 80 % of the length of the feature, were used for parallelism and perpendicularity measurements. The expanded uncertainty of a length measurement on our CMM is 0.0034 mm over 450 mm of travel.

#### 8.2.2 Surface Roughness

Surface roughness was measured using a stylus profilometer, but an optical surface profilometer would be equally as effective. The reason the stylus profilometer was preferred was that the surface roughness could be measured over a long range with a very large number of points. The surface roughness was measured along a 25 mm line of the top surface next to the ramp feature, collecting a point every 0.25 μm. Certainly modern optical surface profilometers are capable of collecting data over that same long range, but they cannot do so in one measurement; they do so by stitching several measurements together. This stitching adds to the uncertainty of the measurement.

The importance of the cutoff value in reporting the surface roughness results should be noted. A long cutoff of 2.5 mm was used for our measurements. This value is larger than the 0.8 mm cutoff typically used for machined surfaces. This value was chosen after examining the raw surface profile with experts in surface roughness measurement and observing a periodicity to the surface that would be filtered out with a 0.8 mm cutoff. If a cutoff of 0.8 mm was used, the surface roughness value would be smaller, but it would be less characteristic of the actual surface.

### 8.3 Additional/Alternative Measurements

**Fig. 10:**
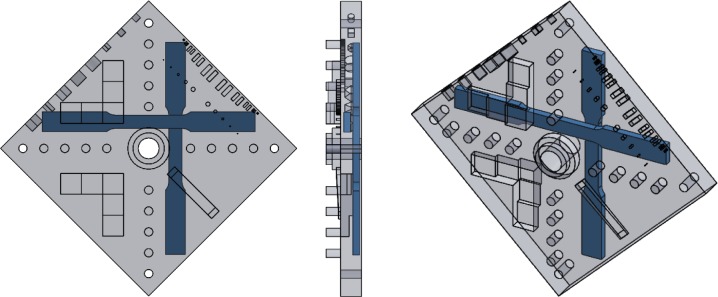
Top view (left), right-side view (center) and oblique view (right) showing an example of the positions from which XY and YX tension testing specimens can be extracted [47].

Because the test artifact is designed, and should be built, as a solid part, several additional tests regarding the physical and mechanical properties of the test artifact can be conducted. There are several tests that can assess the porosity of a part or test specimen and those tests can be accommodated with the proposed test artifact design. Ultrasonic wave testing should be conducted vertically through the top step of the positive staircase or horizontally through the part from the reference side (datum feature H in Fig. 8) toward the opposite side. These features are chosen as targets because they offer the longest travel of the ultrasonic wave and therefore the greatest chance for successful measurement (the 17 mm from the top of the positive staircase to the bottom of the part should provide sufficient wave propagation even if no porosity is present). For tests that require smaller specimens (e.g., X-ray tomography) the pins can be removed from the test artifact and examined. Further, tension testing specimens can be extracted from the bulk of the part in both the x- and y-directions to check for any lateral anisotropy. Figure 10 depicts an example showing from where the tension testing specimens can be extracted through machining or other removal methods.

## 9. Results

**Fig. 11:**
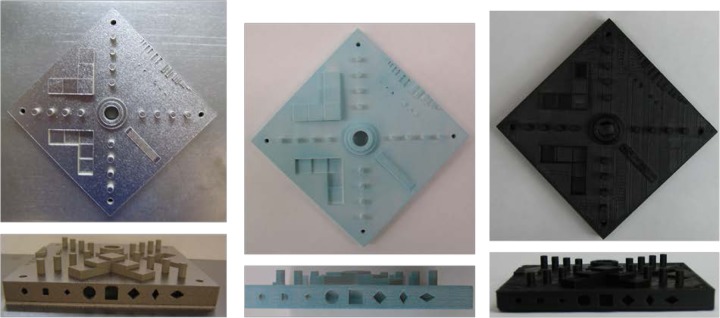
Photographs of the test artifact as built by PBF (stainless steel), binder jetting, material extrusion, respectively from left to right.

The AM test artifact was first built in starch and in polymer before being built in stainless steel. The starch test artifact was built using a binder jetting system and the polymer test artifact was built using a material extrusion system. These builds served as proof of concept before the longer, more expensive stainless steel build. The binder jetting system took 1 hour to build the part (followed by a 1 hour drying period) and the material extrusion system took 3.5 hours to build, though this polymer part was not solid throughout. The stainless steel test artifact was built using a PBF-laser system and took 18 hours to build. Figure 11 shows photographs of the parts built on these three systems. The top surface of the stainless steel part has an average roughness (R_a_) of 5.56 μm and a maximum roughness height (R_t_) of 43.89 μm.

### 9.1 DMLS™ Processing Parameters

It should be noted here that build times are partially a result of the processing parameters used to manufacture the part. Since different materials may require different processing parameters, the build time may vary for parts built in different materials, even on the same system. In general, one set of processing parameters were used to manufacture the stainless steel parts that are the focus of this section. There are four exposure types: two contour exposures (one before other exposures, the pre-contour, and one after other exposures, the post contour), a skin exposure, and a core exposure. The thickness of the skin is set by a processing parameter, which was set to 4.00 mm (both vertically and horizontally) for our processing parameter set. Each exposure type uses different strategies and different settings. Therefore, to fully describe the exposure parameters used to manufacture a part, the parameters for each exposure type must be fully defined.

The very first area exposed on each layer is the pre-contour. For this exposure, the laser is focused on the outer edge of the part, and the laser beam traces the perimeter of the part. The pre-contour is either one pass around the perimeter, or two passes around the perimeter with the beam separated by a certain thickness. Our processing parameter set utilized two pre-contour passes, the first offset from the edge by 0.080 mm, the second offset by 0.040 mm, both using a laser power of 60 W and a scan speed of 700 mm/s. No special strategy was used for fine features.

**Fig. 12:**
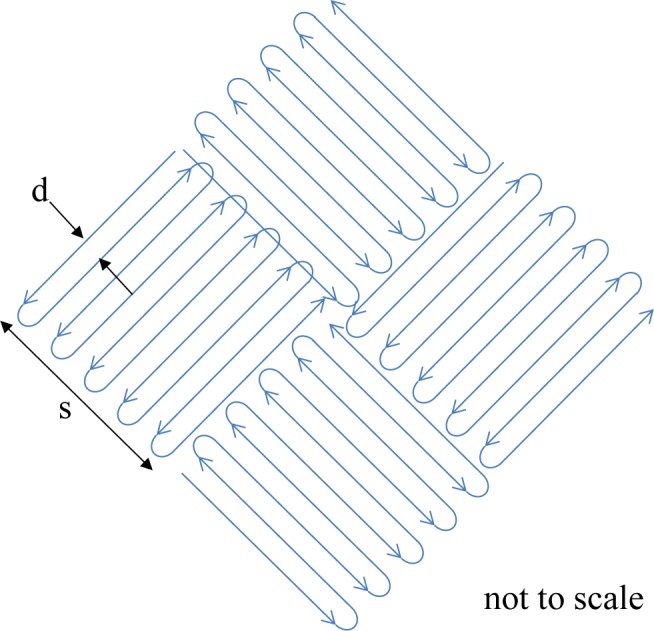
Schematic of the core exposure pattern.

Following the pre-contour, the laser beam is defocused and the core of the part is exposed. For our process parameter set, the core of the part is exposed in a checker-board or basket-weave fashion with neighboring squares (see Fig. 12). The laser rasters very quickly back and forth, making one square at a time. The direction of rastering for each neighboring square is perpendicular to the adjacent squares. For our parameter set, the squares are 10 mm wide (s in Fig. 12). While the beam is rastering within each square, the power is 195 W, the scan speed is 1000 mm/s, and the distance between raster lines, d, is 0.10 mm. Each square overlaps by 0.05 mm. The checker-board pattern is rotated by 45 degrees each layer to minimize anisotropy.

**Fig. 13:**
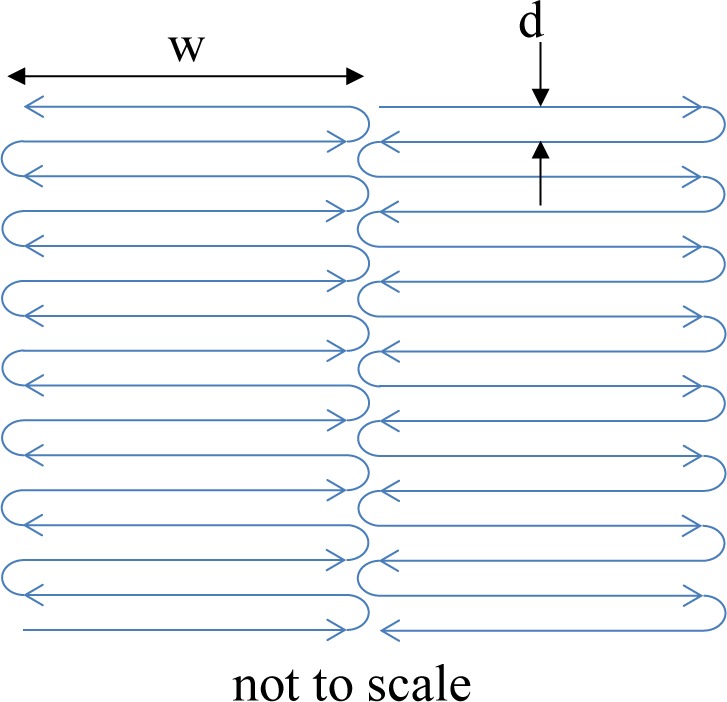
Schematic of skin exposure pattern.

The skin is exposed in a striped pattern. A stripe is merely the rastering of the laser beam back and forth as it progresses across the part (see Fig. 13). For our parameter set, the stripe width, w, is 10.0 mm, and neighboring stripes overlap by 0.05 mm. There are three types of skin, the “sideskin” is measured laterally from the edge of the part. The “upskin” are the top few layers above which there will be no sintered material. The “downskin” is the bottom few layers, below which there is no exposed material. For the sideskin, the beam power is 195 W, the beam scan speed is 1000 mm/s, and neighboring raster lines are separated by 0.10 mm, d. The stripe pattern is offset from the part’s nominal contour by 0.04 mm. For the downskin, the power is 195 W, scan speed is 3000 mm/s, and neighboring raster lines are separated by 0.04 mm. The thickness of the downskin is set to 0.06 mm, thus the downskin exposure is only used on the bottom three layers of downward facing surfaces. For the upskin, the power is 160 W, scan speed is 500 mm/s, and neighboring raster lines are separated by 0.10 mm. The thickness of the upskin is set to 0.02 mm, thus the upskin exposure is only used on the top layer of upward facing surfaces. Similar to the core exposure, the directions of the stripes are rotated by 45° at each layer.

Often, the laser beam is not turned on throughout the entire rastering pattern. A typical rastering pattern has the beam following a serpentine path as it progresses along the part. Because this serpentine pattern has curved portions, the beam will have periods of acceleration and deceleration as it follows the curved path. If “skywriting” is turned on, the laser beam will be switched off during the curved (accelerating and decelerating) portions and will only be on for the linear, constant speed portions. For our parameter set, skywriting is turned on for both the skin and the core exposures.

The very last area exposed on each layer is the post-contour. This exposure is very similar to the pre-contour. Our processing parameter set utilized two post-contour passes, the first offset from the edge by 0.020 mm, the second offset by 0 mm, both using a laser power of 60 W and a scan speed of 700 mm/s. A special strategy was used for fine features. For sharp corners and features smaller than the beam diameter, the beam path is a straight line down the center of the feature. The edge factor for this strategy was set to 2.0, meaning the beam path will remain 2x the beam diameter away from a corner or fine feature edge.

### 9.2 Repeatability

**Fig. 14:**
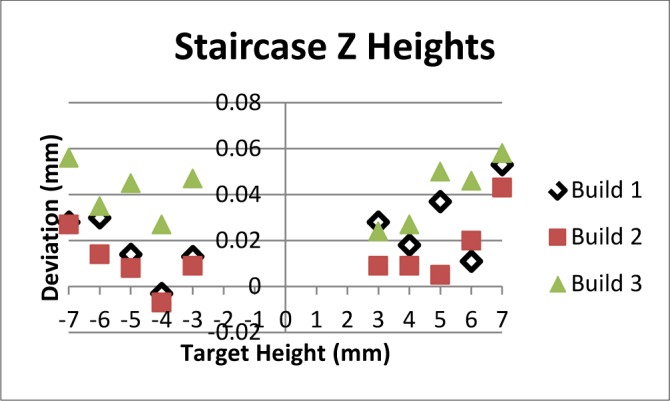
Results of z-height measurement on positive and negative staircase features for three consecutive builds.

**Fig. 15:**
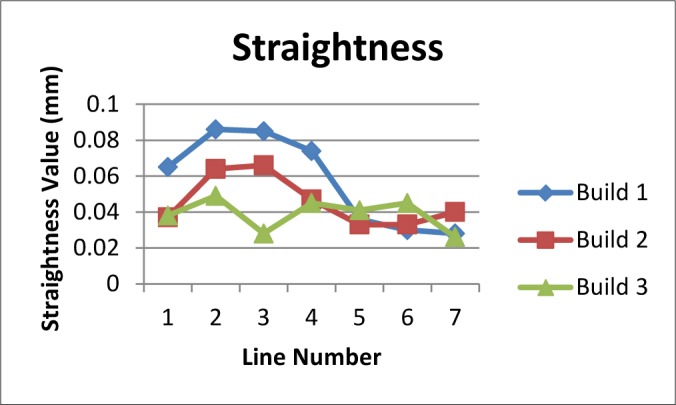
Results of straightness measurements on the long vertical surfaces of the positive and negative staircase features as well as the three outer edges of the test artifact for three consecutive builds.

**Fig. 16:**
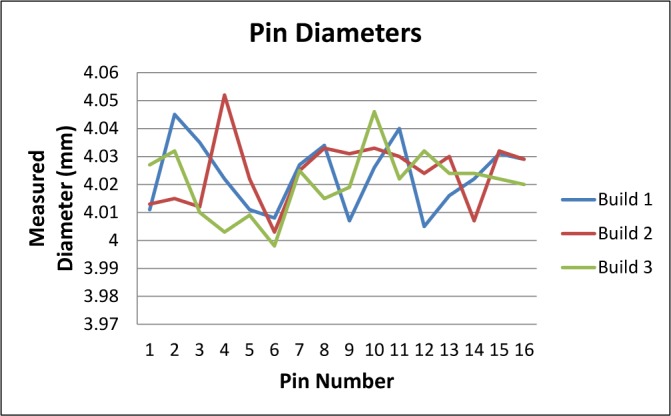
Pin diameters for three consecutive builds. Note that all pins were larger than specified.

**Fig. 17:**
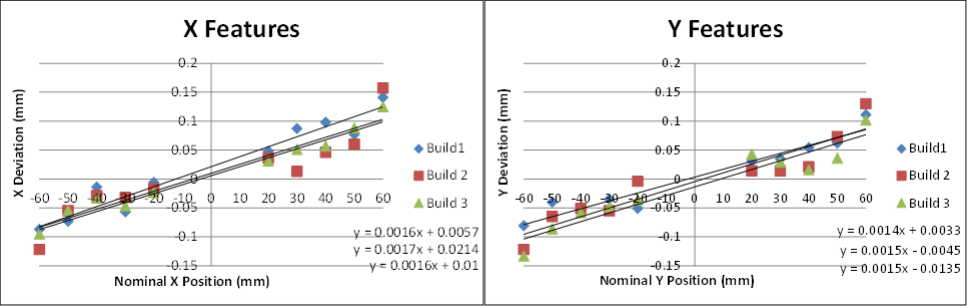
Position deviation (nominal minus measured) of the pins and holes aligned with the AM machine’s x-axis direction (left) and y-axis direction (right). Note that all positions deviated toward the center of the part.

Following the proof-of concept builds, the test artifact was built three consecutive times in stainless steel on the same system without making any changes to processing parameters. Using the results of several of the measurements (staircase z-heights, straightness measurements, pin/hole position and diameter, top surface flatness), the average of the standard deviations was calculated as a measure of repeatability of the system. The calculated repeatability (two times the average standard deviation) of 30 μm agreed with the system manufacturer’s specification. Plots of the various measurements can be seen in Figs. 14–17.

### 9.3 System Improvement

**Fig. 18:**
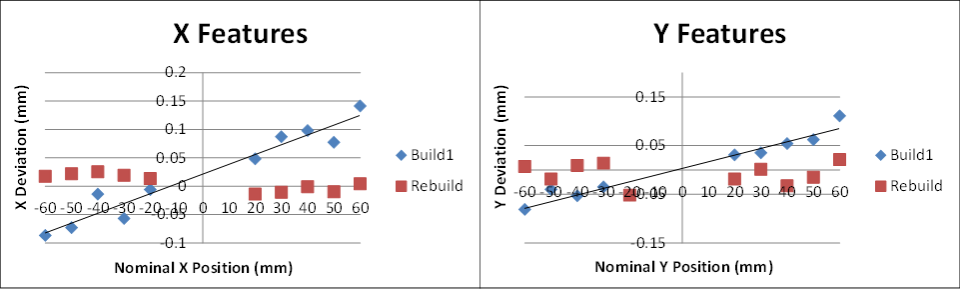
Position deviation (nominal minus measured) of the pins and holes aligned with the AM machine’s x-axis direction (left) and y-axis direction (right) both before and after adjustment to the axis scaling machine setting.

The AM system used in this study allows the user to adjust machine settings for x- and y-axis scaling as well as beam offset. As seen in Fig. 17, the pin and hole positions all deviated toward the center of the part. This is a result of inaccurate axis scaling. By calculating the slope of a line fit to the pin/hole positions, the user can determine the change needed in scaling. The slopes of the lines fit to the pins in the x-direction (0.0016) and in the y-direction (0.0014) for build 1 were converted to percentages and added to the original scaling values. These new values were input into the machine and the part was rebuilt. Figure 18 demonstrates the improvement in the positions of the pins and holes achieved by adjusting the axis scaling. The rebuilt test artifact had only one pin position that deviated by more than the repeatability of the system.

Similarly, the measurements on the test artifact demonstrate an inaccurate machine setting for beam offset and allow the user to calculate an improved setting. Figure 16 shows that all pin diameters in the original builds were too large. The average pin diameter for build 1 was 4.023 mm. All of the holes in build 1 were too small, averaging 3.885 mm. A new beam offset was calculated by taking the deviation from nominal of the average pin diameter and the average hole diameter, averaging the two values, and dividing by two. As such, the value for beam offset was increased by 0.034 mm and the test artifact was rebuilt (note that the scaling and offset were both changed at the same time and the test artifact was only rebuilt once). The average pin diameter on the rebuilt test artifact was 3.937 mm and the average hole diameter on the rebuilt test artifact was 3.985 mm.

An improved value for beam offset can be determined by iteratively calculating the new beam offset and the new axis scalings. For example, the user can calculate new values for axis scaling and beam offset, and rather than input those values directly into the machine, the user can re-calculate the beam path used to generate various features and thereby calculate a theoretical new size for those features. The axis scaling and beam offset can then be re-calculated based on any deviation from this theoretical new feature size from the nominal feature size. The holes, which figure into half of the beam offset calculation, are further from center than the pins and therefore are more affected by changes in the axis scaling. This iterative approach may be more important when large changes in beam offset and scaling are required.

While the focus here is on adjusting machine settings for a laser-based PBF system, other systems (even without laser beams) often have similar adjustable machine settings. For example, a binder jetting system may have a setting for “bleed compensation.” This setting compensates for the amount the binder may wick into neighboring powder particles, thus affecting part geometry. An error in bleed compensation would manifest itself similarly to an error in beam offset, and a new value for that compensation can be calculated similarly. Other systems may not have compensation for axis scaling, but many linear drive and motor controllers allow the input of axis position compensation tables that would be analogous to axis scaling.

### 9.4 Ramp Feature

**Fig. 19:**
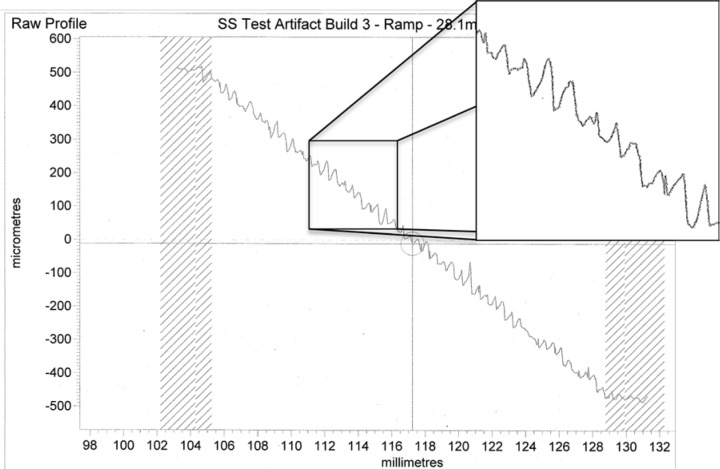
Surface profile of the complete ramp feature. Inset shows zoomed area that demonstrates peaks toward right side of each step.

**Fig. 20:**
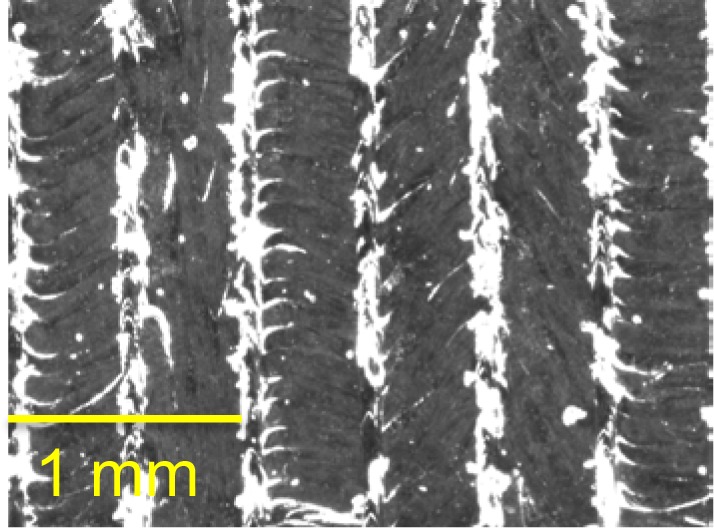
Optical micrograph of a small portion of the ramp feature. The lighter colored portions are the edges of each individual step in the ramp.

The ramp feature indeed shows the degree of stairstepping expected of a build with 0.020 mm layers. Fifty distinct steps are clearly visible on the artifact along the ramp profile. A surface profile of the entire ramp feature (seen in Fig. 19), reveals a more ambiguous situation. The form of the profile does not show clean, flat steps. The surface profilometer proceeded from the top of the ramp to the bottom of the ramp (from left to right in the figure). Some degree of rounding and sloping was expected as the stylus stepped down from one step to the next because of the cone angle of the stylus profilometer. This rounding would be an effect of the measurement system. However, the peaks toward the ends of the steps (on the right edge of each step) were not expected and appear to be truly representative of the surface and not an effect of measurement. A micrograph of a section of the ramp, shown in Fig. 20, shows evidence of some type of edge effect on the ramp steps. These peaks make quantifying the step heights very difficult.

### 9.5 Lateral Features

**Fig. 21:**
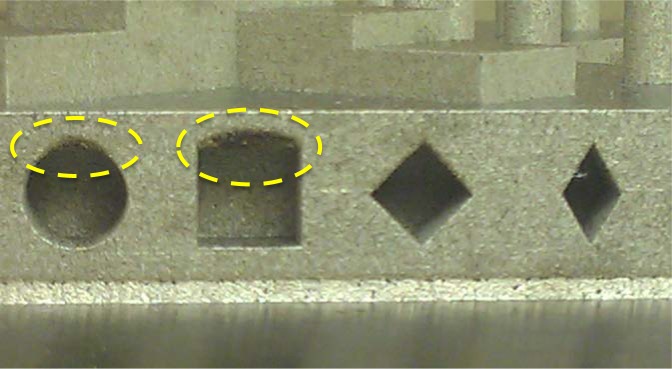
Photograph of the lateral features with the poorly formed surfaces highlighted.

Many of the lateral features (produced without support structures) show poorly formed upper surfaces. Unfortunately, the features were inaccessible for the CMM probe, so the analysis is purely observational. However, Fig. 21 shows clear evidence of the poorly formed surfaces. The most obvious evidences is the singed (blackened) and curved upper surface of the feature intended to be square. There is similar (though lesser) singeing on the top of the 6 mm cylindrical features. The diamond features with upper half-angle of 45 degree and 30 degree angles do not show the evidence of singeing, but the diamond feature with the 60 degree upper half-angle (not pictured) does show singeing. This leads to a DMLS design rule for stainless steel builds that features forming an angle of less than 45 degrees relative to the build platform may not need support structures.

The downward facing surfaces in these lateral features are poorly formed, showing evidence of spalling and singeing (as seen by the dark regions in Fig. 21). This is a result of the different thermal conductivity of the underlying powder compared to underlying solid material. This difference results in a change in the size and temperature of the melt pool in the areas with underlying powder. Better control of the processing parameters in these areas allows the melt pool to remain more consistent and these surfaces can be manufactured more accurately [49].

### 9.6 Minimum Feature Size

**Fig. 22:**
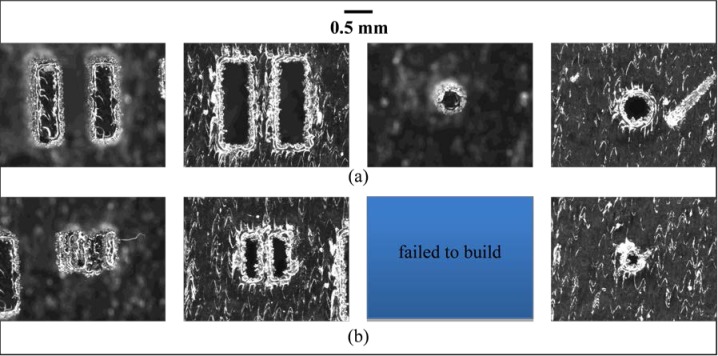
Optical micrographs of the fine features, (a) 0.5 mm features and (b) 0.25 mm features. From left to right: neighboring rectangular bosses, neighboring rectangular holes, pin, hole.

Figure 22 shows optical micrographs of several of the fine features revealing that the minimum feature size achievable on this system in stainless steel is between 0.5 mm and 0.25 mm. It is clear from these micrographs that the 0.5 mm features were all fully formed. On the other hand, several of the 0.25 mm features either failed to build or were clearly built badly. This agrees with the system specification of a minimum feature size of 0.3 mm.

The material is noted along with the minimum feature size result because the two may be correlated. Minimum feature size is likely a function of the size of the heat affected zone, the positioning accuracy of the laser beam positioning system, and the beam width compensation. All of these are material dependent.

Note that the edge phenomenon discussed in Sec. 9.4 can be seen here as well. This edge phenomenon makes dimensional measurement of these fine features difficult. Dimensional measurement from optical micrographs is reliant on edge detection algorithms. The edge phenomenon observed in these micrographs makes the determination of the feature’s actual edge ambiguous. For example, it is unclear if the dimension of the 0.5 mm pin should be defined as the dark portion in the middle of the micrograph or the outer edge of the lighter material surrounding the center portion. Because of this ambiguity, dimensional measurement on these features was not done despite the fact that our calibrated optical system would allow it.

### 9.7 Other AM Technologies

**Fig. 23:**
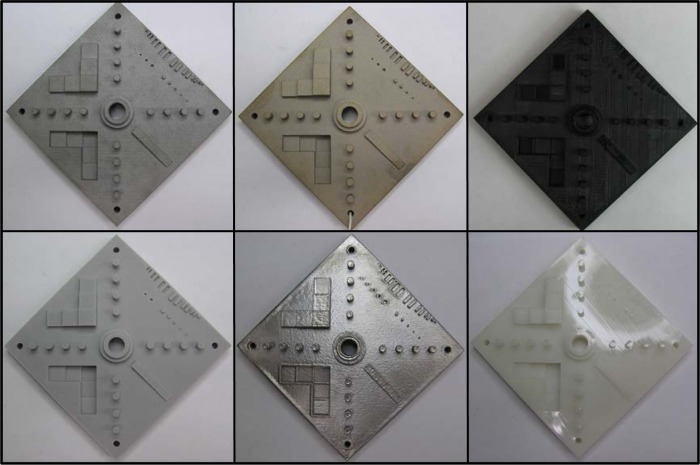
Photographs of the test artifact as built by collaborators. Clockwise from top left: PBF (stainless steel by selective laser melting), binder jetting, material extrusion (FDM), vat photopolymerization (SLA), PBF (titanium by electron beam melting), and PBF (polymer by SLS).

The design of the AM test artifact was shared with several collaborators in industry, government, and academia. Some of these collaborators have built the test artifact with systems they own or were evaluating for purchase. Figure 23 shows the results of some of these builds in different materials by different AM systems. The focus of this paper is not on a comparison of different AM systems or technologies, and further, the design was shared with collaborators with minimal instruction, and the collaborators delivered their built test artifacts with varying levels of accompanying detail. Therefore, the results of measurements taken on these test artifacts will not be discussed. However, this varying level of detail demonstrates the need for standardization of not just the test artifact design, but also the detailed reporting of the build processing parameters and measurement results.

## 10. Standardization

We believe that standards will be broadly used by an ever-expanding group of vendors and users of AM technology and AM products. These standards, especially in the area of performance characterization, will be utilized by users to better understand their particular systems as well as the process and technologies in general. This improved understanding will help users to make buy/sell decisions and better allocate resources, define and maintain system capabilities, conduct manufacturability analyses, and optimize system performance. Further, standards will facilitate the use of AM parts in critical systems, especially in the aerospace and medical industries, by linking part and system data to well characterized materials and processes. Currently, the primary standards development organization promoting AM standards is ASTM International Committee F42 on Additive Manufacturing Technologies. NIST is leading efforts for strategic planning of the F42 committee, which has developed an overall hierarchy for standards. A standards strategy for the AM test artifact aligns well with this hierarchy.

We believe that one artifact geometry will be sufficient to characterize all types of AM systems. All systems must be able to make parts that have features that conform to GD&T standards, and all systems should conform to a standard coordinate system [47]. As such, one standard at the highest level of the hierarchy will suffice to describe the significance and use of the test artifact and associated measurement results, the general description of the geometry and important features in the test artifact, the measurements to be taken on the test artifact, as well as the requirements for reporting testing results. Since this performance characterization will produce quantitative results, and the standard should be the highest level in the hierarchy, this standard should be a Test Method.

However, the seven standard AM process categories can be very different [1], and machines for each of these technologies can vary in size and pertinent processing parameters. For this reason, the instructions and details for manufacturing the test artifact should be described at a lower level in the hierarchy, the process category level. There may be seven separate Standard Practice documents describing how to build the AM test artifact, one for each process category. These standard practices would provide links for users to download one of possibly several versions of the AM test artifact; different systems might need a range of sizes or scales for the test artifact and its features. Allowing download of the files would avoid inaccuracies and inconsistencies that might arise if each user generated the design using their own software. The standard practices would provide detailed information on any pre-process design file manipulations, procedures for preparing the machine for building, and location and orientation of the test artifact within the build volume. The standard practices would not dictate specific processing parameters to use (that would be up to the user or agreed upon by the purchaser and supplier), but will detail how the processing parameters should be reported. It is important that these parameters be reported in such a manner that another user can examine the report and duplicate the build as closely as possible on a similar or different machine.

The AM test artifact, as described in Secs. 7 and 8, has been introduced as a Test Method to the ASTM F42 subcommittee on Test Methods. The work item, WK40419, proposes a new standard titled “Performance evaluation of additive manufacturing systems through measurement of a manufactured test piece.” In addition, standardization of an AM test artifact was recently chosen as a top priority for joint development by ASTM F42 and the ISO Technical Committee 261 on Additive Manufacturing. The standardization procedure will likely generate more feedback, which may result in slight (or possibly significant) modifications to the AM test artifact design. Some preliminary feedback related to providing more detail for inspection and extracting mechanical testing specimens has already been incorporated. Other recommendations to improve the design and the design files are being considered, including non-uniform spacing of pins/holes to capture periodic positioning error, longer datum surfaces for ramp feature to ease surface profile data analysis, and incorporating GD&T into the part model.

## Figures and Tables

**Table 1:** The features and characteristics investigated on the test artifact

Characteristics investigated	Feature(s) used to demonstrate
Straight features	Vertical walls of staircases; outer edges
Parallel or perpendicular features	Vertical walls of staircases; outer edges
Circular or arced features	Center hole; central cylinders
Concentric circles or arcs	Central cylinders
Fine features	Fine features, holes, and pins
3D or freeform features	Ramp; lateral features
Holes and bosses	4 mm pins and holes; center holes and central cylinders; staircases; fine features
Multiple planes	Lateral features
Location	4 mm pins and holes
Geometric errors of laser positioning axes	4 mm pins and holes
Geometric errors of build platform	Staircases; center hole; ramp
Alignment errors between axes	Top surface and center hole
Errors in beam size	4 mm holes and pins

## Footnotes

1 Official contribution of the National Institute of Standards and Technology (NIST); not subject to copyright in the United States. The full descriptions of the procedures used in this paper require the identification of certain commercial products. The inclusion of such information should in no way be construed as indicating that such products are endorsed by NIST or are recommended by NIST or that they are necessarily the best materials, instruments, software, or suppliers for the purposes described.

## References

[1] ASTM Standard F2792 (2012a). Standard Terminology for Additive Manufacturing Technologies.

[2] Richter J, Jacobs P (1992). Accuracy, in: Rapid Prototyping & Manufacturing, P Jacobs.

[3] Campanelli SL, Cardano G, Giannoccaro R, Ludovico AD, Bohez ELJ (2007). Statistical analysis of the stereolithographic process to improve the accuracy. Computer-Aided Design.

[4] Byun H-S, Lee K, Kumar V, Gavrilova M, Tan C, L’Ecuyer P (2003). Design of a New Test Part for Benchmarking the Accuracy and Surface Finish of Rapid Prototyping Processes. Computational Science and Its Applications — ICCSA 2003.

[5] Kruth J-P, Vandenbroucke B, Vaerenbergh JV, Mercelis P (2005). Benchmarking of different SLS/SLM processes as rapid manufacturing techniques.

[6] Scaravetti D, Dubois P, Duchamp R (2008). Qualification of rapid prototyping tools: proposition of a procedure and a test part. The International Journal of Advanced Manufacturing Technology.

[7] Shellabear M (1998). Model manufacturing processes–State of the art in rapid prototyping.

[8] ASME B5.54 (2005). Methods for Performance Evaluation of Computer Numerically Controlled Machining Centers.

[9] ISO IS 10791-7: 1998 (1998). Test conditions for machining centers–Part 7: Accuracy of a finished test piece.

[10] NAS (1969). 979 Uniform Cutting Tests--NAS series metal cutting equipment specifications.

[11] Cooke AL, Soons JA Variability in the Geometric Accuracy of Additively Manufactured Test Parts.

[12] Kruth JP (1991). Material Incress Manufacturing by Rapid Prototyping Techniques. CIRP Annals – Manufacturing Technology.

[13] Lart G Comparison of Rapid Prototyping Systems.

[14] Childs THC, Juster NP (1994). Linear and Geometric Accuracies from Layer Manufacturing. CIRP Annals – Manufacturing Technology.

[15] Jayaram D, Bagchi A, Jara-Almonte CC, O’Reilly S (1994). Benchmarking of Rapid Prototyping Systems – Beginning to Set Standards. Proceedings of the Solid Freeform Fabrication Symposium.

[16] Ippolito R, Iuliano L, Gatto A (1995). Benchmarking of Rapid Prototyping Techniques in Terms of Dimensional Accuracy and Surface Finish. CIRP Annals – Manufacturing Technology.

[17] Xu F, Wong YS, Loh HT (2000). Toward generic models for comparative evaluation and process selection in rapid prototyping and manufacturing. Journal of Manufacturing Systems.

[18] Campbell RI, Martorelli M, Lee HS (2002). Surface roughness visualization for rapid prototyping models. Computer-Aided Design.

[19] Grimm T (2003). Rapid Prototyping Benchmark: 3D Printers.

[20] Grimm T (2010). 3D Printer Benchmark: North American Edition.

[21] Mahesh M, Wong Y, Fuh JYH, Loh HT (2004). Benchmarking for comparative evaluation of RP systems and processes. Rapid Prototyping J.

[22] Mahesh M, Wong YS, Fuh JYH, Loh HT (2006). A six-sigma approach for benchmarking of RP&M processes. International Journal of Advanced Manufacturing Technology.

[23] Kim GD, Oh YT (2008). A benchmark study on rapid prototyping processes and machines: Quantitative comparisons of mechanical properties, accuracy, roughness, speed, and material cost.

[24] Brajlih T, Valentan B, Balic J, Drstvensek I (2011). Speed and accuracy evaluation of additive manufacturing machines. Rapid Prototyping J.

[25] Gargiulo EP (1992). Stereolithography process accuracy: user experience.

[26] Bedal B, Nguyen H, Jacobs PF (1996). Advances in Part Accuracy. Stereolithography and Other RP&M Processes.

[27] Geiger M, Coremans A, Neubauer N, Niebling F (1996). Advanced rapid prototyping by laser beam sintering of metal prototypes: design and development of an optimized laser beam delivery system. Proc SPIE.

[28] Dimitrov D, van Wijck W, Schrevel K, de Beer N, Meijer J (2003). An investigation of the capability profile of the three dimensional printing process with an emphasis on the achievable accuracy. CIRP Annals.

[29] Dimitrov D, van Wijck W, Schreve K, de Beer N (2006). Investigating the achievable accuracy of three dimensional printing. Rapid Prototyping J.

[30] Hopkinson N, Sercombe TB (2008). Process repeatability and sources of error in indirect SLS of aluminium. Rapid Prototyping J.

[31] Johnson WM, Rowell M, Deason B, Eubanks M (2011). Benchmarking Evaluation of an Open Source Fused Deposition Modeling Additive Manufacturing System.

[32] Senthilkumaran K, Pandey PM, Rao P (2011). Statistical modeling and minimization of form error in SLS prototyping. Rapid Prototyping J.

[33] Nguyen H, Richter J, Jacobs P (1992). On Windowpanes and Christmas-Trees: Diagnostic Techniques for Improved Part Accuracy, Third Int. Conf Rapid Prototyping.

[34] Castillo L (2005). Study about the rapid manufacturing of complex parts of stainless steel and titanium.

[35] Ning Y, Wong YS, Fuh JYH, Loh HT (2006). An approach to minimize build errors in direct metal laser sintering. IEEE Transactions on Automation Science and Engineering.

[36] Ghany KA, Moustafa SF (2006). Comparison between the products of four RPM systems for metals. Rapid Prototyping J.

[37] Hanumaiah N, Ravi B (2007). Rapid tooling form accuracy estimation using region elimination adaptive search based sampling technique. Rapid Prototyping J.

[38] Pessard E, Mognol P, Hascoet JY, Gerometta C (2008). Complex cast parts with rapid tooling: rapid manufacturing point of view. International Journal of Advanced Manufacturing Technology.

[39] Delgado J, Ciurana J, Reguant C, Cavallini B (2010). Studying the repeatability in DMLS technology using a complete geometry test part.

[40] Pandey PM, Reddy NV, Dhande SG (2003). Improvement of surface finish by staircase machining in fused deposition modeling. Journal of Materials Processing Technology.

[41] Armillotta A (2006). Assessment of surface quality on textured FDM prototypes. Rapid Prototyping J.

[42] Es-Said OS, Foyos J, Noorani R, Mendelson M, Marloth R, Pregger BA (2000). Effect of layer orientation on mechanical properties of rapid prototyped samples. Mater Manuf Process.

[43] Vega V, Clements J, Lam T, Abad A, Fritz B, Ula N, Es-Said OS (2011). The Effect of Layer Orientation on the Mechanical Properties and Microstructure of a Polymer. Journal of Materials Engineering and Performance.

[44] Smith R (1992). Standard Test-Files for Benchmarking RP Systems.

[45] Jurrens KK (1999). Standards for the Rapid Prototyping Industry. Rapid Prototyping J.

[46] ISO IS 841:2001 (2001). Industrial automation systems and integration–Numerical control of machines–Coordinate system and motion nomenclature.

[47] ASTM Standard F2921-11 (2011). Standard Terminology for Additive Manufacturing–Coordinate Systems and Test Methodologies.

[48] Kruth JP, Mercelis P, Van Vaerenbergh J, Craeghs T (2008). Feedback control of selective laser melting.

[49] Craeghs T, Bechmann F, Berumen S, Kruth JP (2010). Feedback control of Layerwise Laser Melting using optical sensors. Physics Procedia.

